# Structural basis for the extended-spectrum antimicrobial activity of Garvieacin Q

**DOI:** 10.1128/aem.01773-25

**Published:** 2026-01-21

**Authors:** Jinsong Duan, Dan Li, Yuqing Zhao, Jiawei Wang

**Affiliations:** 1 State Key Laboratory of Membrane Biology, Beijing Frontier Research Center for Biological Structure, School of Life Sciences, Tsinghua University 98441, Beijing, People's Republic of China; Anses, Maisons-Alfort Laboratory for Food Safety, Maisons-Alfort, France

**Keywords:** antibiotic resistance, antimicrobial peptides, mannose phosphotransferase system, Man-PTS, bacteriocins, non-pediocin-like/class IId bacteriocins, Garvieacin Q, GarQ

## Abstract

Class IIa and IId bacteriocins are antimicrobial peptides with potential for combating antibiotic-resistant pathogens. However, their species-specific activity, dictated by recognition of the mannose phosphotransferase system (Man-PTS) receptor, often restricts their spectrum. Garvieacin Q/Garvicin Q (GarQ), a newly identified class IId bacteriocin, is unusual in that it targets both *Lactococcus garvieae* and the non-lactococcal pathogen *Listeria monocytogenes*, yet the structural basis for this cross-species activity has remained unclear. Using cryo-electron microscopy, we determined the structures of GarQ bound to Man-PTS receptors from *Lactococcus garvieae* and *Listeria monocytogenes*. In *Lactococcus garvieae*, the receptor contains a unique Tudor-like γ+ domain that sterically constrains the N terminus of incoming bacteriocins, thereby enforcing specificity for GarQ while excluding others such as lactococcin A (LcnA). In *Listeria monocytogenes*, GarQ engages the receptor through the same conserved binding mode, effectively bypassing the unusual species barrier. We further demonstrate that the C-terminal length of GarQ is a critical determinant of pore size and target specificity. Together, these findings uncover the structural mechanism underlying GarQ’s atypical extended-spectrum activity and provide a framework for engineering bacteriocins with customized spectra to combat specific pathogens.

**IMPORTANCE:**

This study establishes a structural basis for how the extended-spectrum bacteriocin Garvieacin Q (GarQ) circumvents the canonical species-specificity of class II bacteriocins by engaging mannose phosphotransferase system receptors from different bacterial genera through both conserved and divergent binding modes. We identify a previously unknown Tudor-like γ+ domain in the *Lactococcus garvieae* receptor that sterically restricts the access of other bacteriocins, thereby defining bacteriocin specificity. Moreover, we demonstrate that the C-terminal length of GarQ critically determines pore size and bacterial targets, revealing an engineerable principle for designing synthetic bacteriocins with customized spectra against clinically relevant pathogens.

## INTRODUCTION

The escalating crisis of antibiotic resistance has intensified the search for alternative antimicrobials, with bacteriocins—ribosomally synthesized antimicrobial peptides produced by bacteria—emerging as promising candidates (1, 2). Among these, class II bacteriocins from gram-positive bacteria, particularly the linear, non-post-translationally modified subtypes, have attracted considerable attention for their potent activity and engineering potential (3, 4). This class can be divided into several subgroups: pediocin-like (IIa) bacteriocins, which show strong activity against *Listeria monocytogenes* and also act on closely related genera such as *Pediococcus* and *Enterococcus* (5, 6), and lactococcin-A-like bacteriocins, often grouped within class IId, which are mainly active against *Lactococcus* spp. but display broad-spectrum diversity (7).

A pivotal advance was the discovery that these seemingly disparate bacteriocins share a common cellular receptor, the mannose phosphotransferase system (Man-PTS) (8–10). While central to sugar import (9), the Man-PTS complex has been co-opted as a receptor for diverse agents, including the bacteriophage λ (11) and bacteriocins (12). Specific extracellular loops of its membrane-bound subunits, ManY and ManZ, dictate bacteriocin recognition (13). For example, the α-region of ManY is a primary determinant for binding either IIa or IId bacteriocins (14), whereas the γ-region of *Listeria monocytogenes* ManZ further modulates this interaction (15). This receptor-specificity paradigm has provided a clear explanation for the distinct spectra of class IIa/IId bacteriocins.

The discovery of Garvieacin Q (GarQ), a novel bacteriocin from *Lactococcus garvieae* (BCC 43578), challenges this paradigm (16). Although originally classified as a class IId bacteriocin, GarQ belongs to the subgroup of lactococcin-A-like bacteriocins (including LcnA, LcnB, and LcnZ), which exhibit considerable heterogeneity: LcnA represents a narrow-spectrum, for example, active primarily on *Lactococcus* spp., whereas GarQ displays an extended spectrum across multiple genera, including over 30 *Lactococcus* strains, 6 *Enterococcus* strains, and other sensitive genera such as *Carnobacterium, Pediococcus,* and *Leuconostoc* (17). In both *Lactococcus garvieae* and the non-lactococcal pathogen *Listeria monocytogenes*, the receptor was confirmed to be Man-PTS, with the *Lactococcus garvieae* complex featuring a previously uncharacterized extracellular region, termed γ+ (18, 19). GarQ thus poses a fundamental mechanistic question: how can a single bacteriocin circumvent the precise structural determinants of species specificity to act on two distinct Man-PTS receptors?

To address this, we used high-resolution cryo-electron microscopy (cryo-EM) to determine the structures of the *Lactococcus garvieae* Man-PTS (lgYZ) alone and in complex with GarQ, as well as GarQ bound to the *Listeria monocytogenes* Man-PTS (lmYZ). These structures uncover the role of the unique γ+ domain and reveal an unprecedented mechanism by which GarQ bypasses conventional species barriers. Our findings resolve the paradox of GarQ specificity and provide a structural framework for engineering bacteriocins with broadened or tailored activity spectra, directly connecting the structural insights to potential applications.

## RESULTS

### The lgYZ receptor features a unique Tudor-like domain

**Fig 1:**
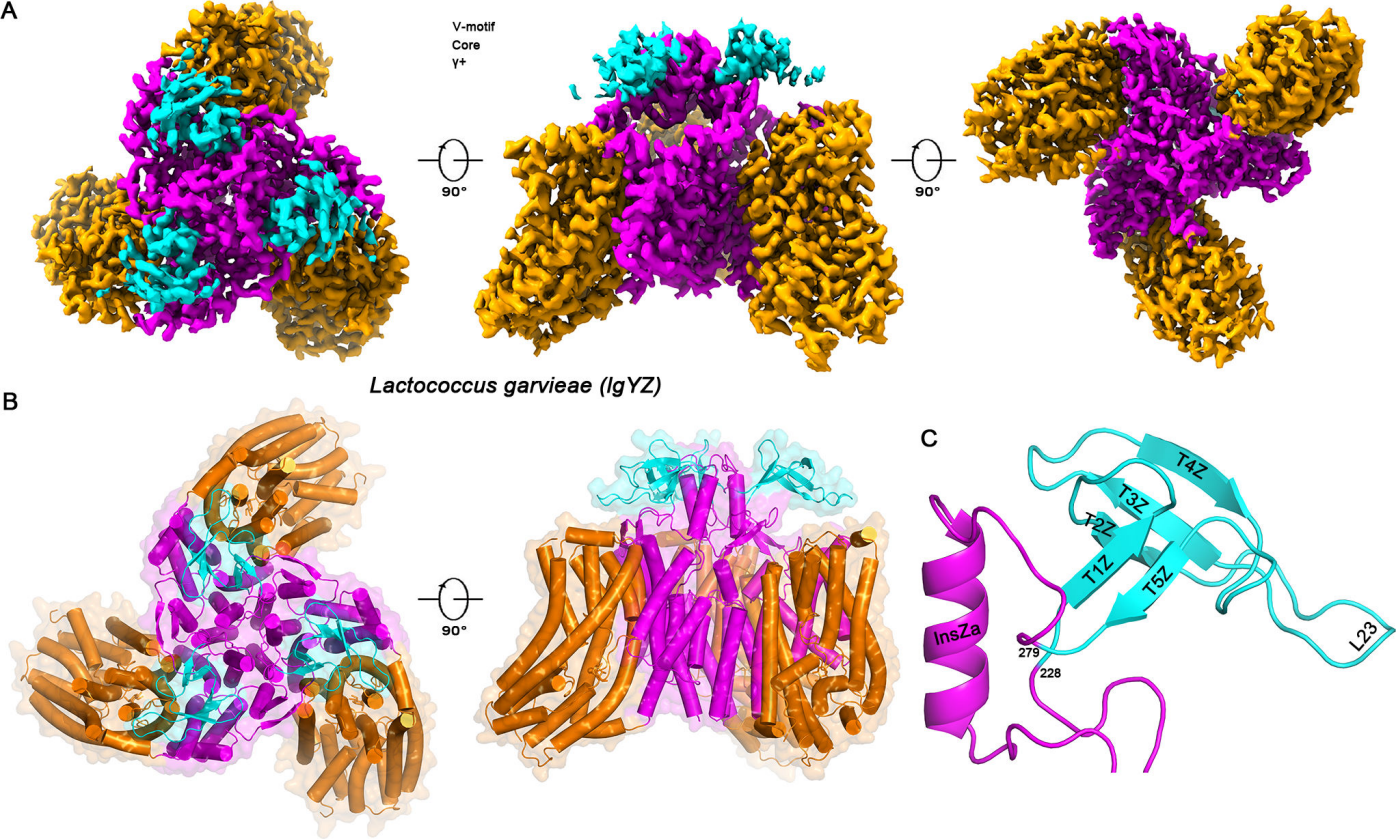
Architecture of the Man-PTS receptor of *Lactococcus garvieae*. (**A**) Cryo-EM reconstruction of the lgYZ complex, displayed from the top, side, and bottom perspectives. Key structural features are colored as follows: V-motif (magenta), Core subdomains (orange), and the unique γ+ region (cyan). (**B**) Top and side views of the atomic model. The top view and side view depict the overall architecture and membrane association, respectively. The lipid bilayer is schematically represented by black lines. (**C**) Close-up view of the γ+ domain insertion, a distinctive structural feature of the *Lactococcus garvieae* ManZ subunit.

C-terminal His-tagged lgManY and lgManZ subunits were co-expressed in *Lactococcus lactis*, purified, and analyzed by single-particle cryo-EM. The resulting 2.81 Å reconstruction (Fig. S1 and Table S1) revealed the canonical Man-PTS transporter architecture (10): a homotrimeric V-motif scaffold surrounded by peripheral Core domains (Fig. 1A and B). Strikingly, within the extracellular V-motif domain, we identified a prominent structural insertion corresponding to the previously annotated γ+ region (18). This element folds into a distinct five-stranded β-barrel (T1Z–T5Z) (Fig. 1C). A DALI search (20) identified this novel fold as a Tudor-like domain (Z-score: 4.2; PDB ID: 6IF4), originally described in the *Drosophila* Tud protein essential for germ cell formation (21).

The presence of this Tudor-like domain on a lactococcal-type receptor presents a structural paradox. Class IIa and IId bacteriocins are typically mutually exclusive in host range, determined by their recognition of distinct α-regions in Man-PTS receptors (4). Class IIa bacteriocins (e.g., pediocin PA-1/PedA) target non-lactococci such as *Listeria* (15, 22), whereas class IId bacteriocins (e.g., LcnA) act specifically on *Lactococcus lactis* Man-PTS (llYZ) receptors carrying a divergent α-region (14). Since *Lactococcus garvieae* possesses a lactococcal-type α-region (Fig. S2), GarQ was predicted to act as a typical class IId bacteriocin. The discovery of a large Tudor-like accessory domain, however, suggested a more complex recognition mechanism that might underlie GarQ’s atypical activity spectrum.

### The γ+ domain dictates bacteriocin specificity through steric restraint

**Fig 2:**
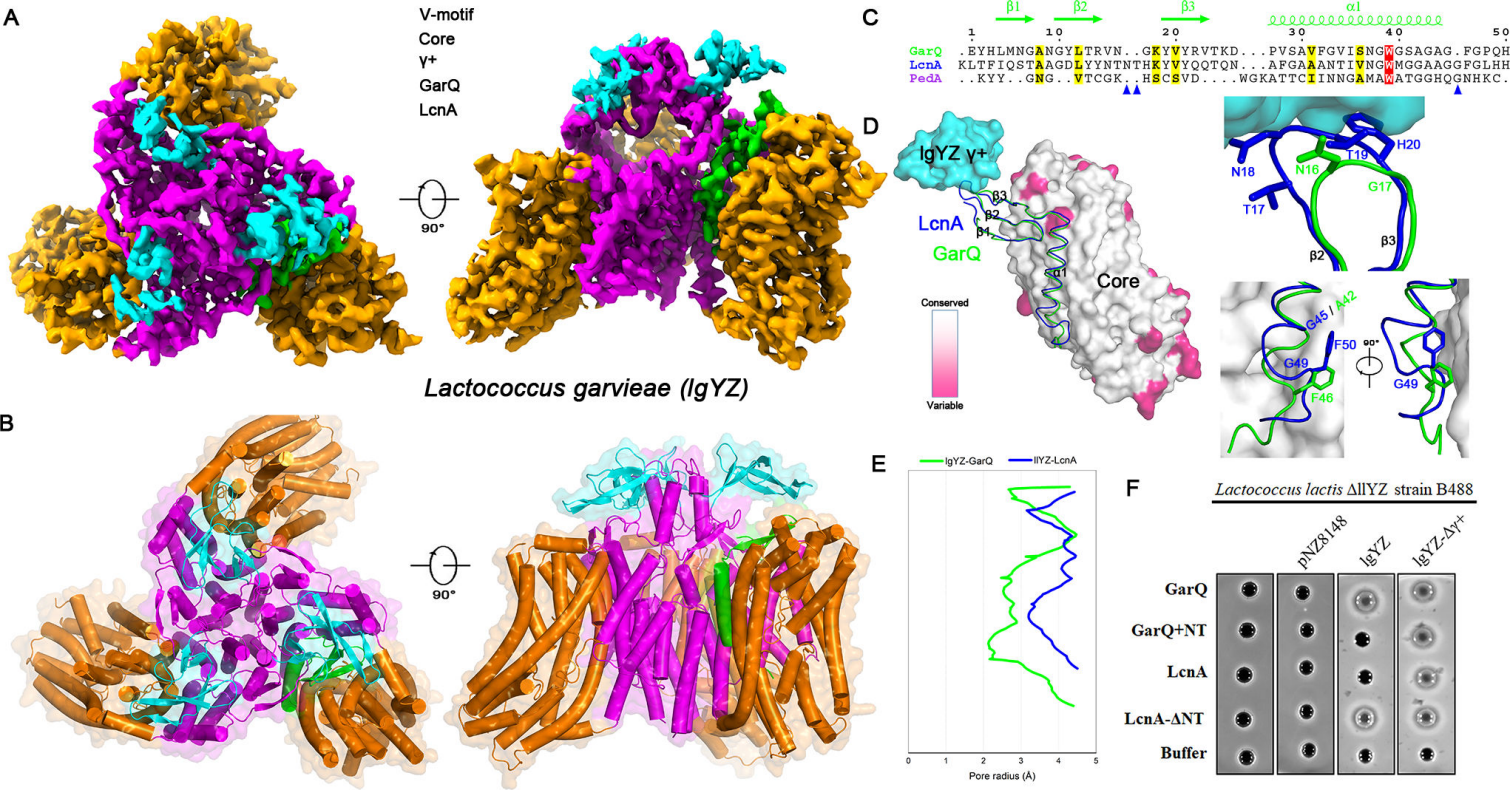
Cryo-EM structure of the Man-PTS receptor of *Lactococcus garvieae* in complex with the bacteriocin GarQ. (**A**) Cryo-EM map of the lgYZ Man-PTS complex bound to GarQ (green), in top and side views. (**B**) Structural model of the trimeric complex (Core: orange; V-motif: magenta) with a single GarQ bacteriocin (green). (**C**) Structure-based sequence alignment of the bacteriocins GarQ, LcnA, and PedA. (**D**) Structural alignment of the GarQ-lgYZ and LcnA-llYZ (PDB ID: 8HFS) complexes, based on their Core domains. The Core domains are colored according to the sequence conservation between the lgYZ and llYZ Man-PTS receptors. The V-motif domains were omitted for clarity to highlight the relative positions of the bound bacteriocins. Top inset: interaction between the N-terminal loop of the bacteriocins (GarQ, green; LcnA, blue) and the region γ+ (cyan) of the lgYZ Man-PTS receptor. Bottom inset: interactions between the C-terminal regions of GarQ and LcnA with the receptor Core domain. (**E**) Pore radii along the central axis of the putative bacteriocin-induced channel for GarQ (green) and LcnA (blue), as computed using HOLE software (23).(**F**) Bactericidal activity of GarQ and LcnA variants against *Lactococcus garvieae*. Control strains include *Lactococcus lactis* B488 (a Man-PTS llYZ deletion mutant) and strains containing the empty vector pNZ8148.

The cryo-EM structure of the GarQ-lgYZ complex (Fig. S3) revealed that GarQ adopts the canonical wedge-like binding mode of class IId bacteriocins, inserting into the Man-PTS to induce pore formation (Fig. 2A and B). Both GarQ and LcnA share a conserved three-stranded N-terminal β-sheet, yet critical structural differences explain their divergent activities (Fig. 2C). Structural alignment of GarQ-lgYZ with the LcnA-llYZ complex (PDB ID: 8HFS) based on the Core domains showed equivalent binding sites (Fig. 2D), but the lgYZ receptor’s Tudor-like γ+ domain imposes a steric restriction on GarQ, constraining its N-terminus (Fig. 2D, top inset). Specifically, the γ+ domain occludes the β2–β3 loop, where LcnA carries a two-residue (NT) insertion absent in GarQ (Fig. 2C). Computational modeling confirmed that introducing the NT insertion into GarQ causes a steric clash with the γ+ domain, explaining its evolutionary exclusion.

A second determinant of pore architecture lies in the C-terminus (Fig. S4). LcnA contains an extra glycine that bends its C-terminal loop, projecting a tyrosine upward toward the Core domain (Fig. 2D, bottom inset). By contrast, GarQ lacks this glycine (Fig. 2C), producing a more extended conformation with the equivalent tyrosine that projects downward. This structural difference requires a wider separation between the V-motif and Core domains in the LcnA-llYZ complex, accounting for its large pore radius relative to the compact GarQ-lgYZ pore (Fig. 2E).

Functional assays validated this steric mechanism (Fig. 2F). A *Lactococcus lactis* ΔllYZ strain B488 expressing lgYZ (B488-lgYZ) became sensitive to GarQ but remained resistant to LcnA, confirming that the γ+ domain confers specificity. Introducing the NT insertion into GarQ (GarQ+NT) abolished the activity, whereas deleting it from LcnA (LcnA-ΔNT) enabled killing of the chimeric strain. Moreover, removing the γ+ domain from lgYZ (B488-lgYZ-Δγ+) rendered the strain fully susceptible to wild-type LcnA. Together, these structural and functional data establish the γ+ domain as a molecular gatekeeper that sterically excludes bacteriocins carrying incompatible structural features.

### A conserved binding mode enables GarQ’s cross-species activity

The species specificity of bacteriocins is classically dictated by their recognition of phylogenetically distinct α-regions within Man-PTS receptors (4). This paradigm enforces mutually exclusive targeting of the following: class IIa bacteriocins (e.g., PedA) act on *Listeria* receptors, while class IId bacteriocins (e.g., LcnA) act on lactococcal receptors (14). GarQ, however, defies this rule by targeting both *Lactococcus garvieae* and *Listeria monocytogenes*, posing a central mechanistic question.

**Fig 3:**
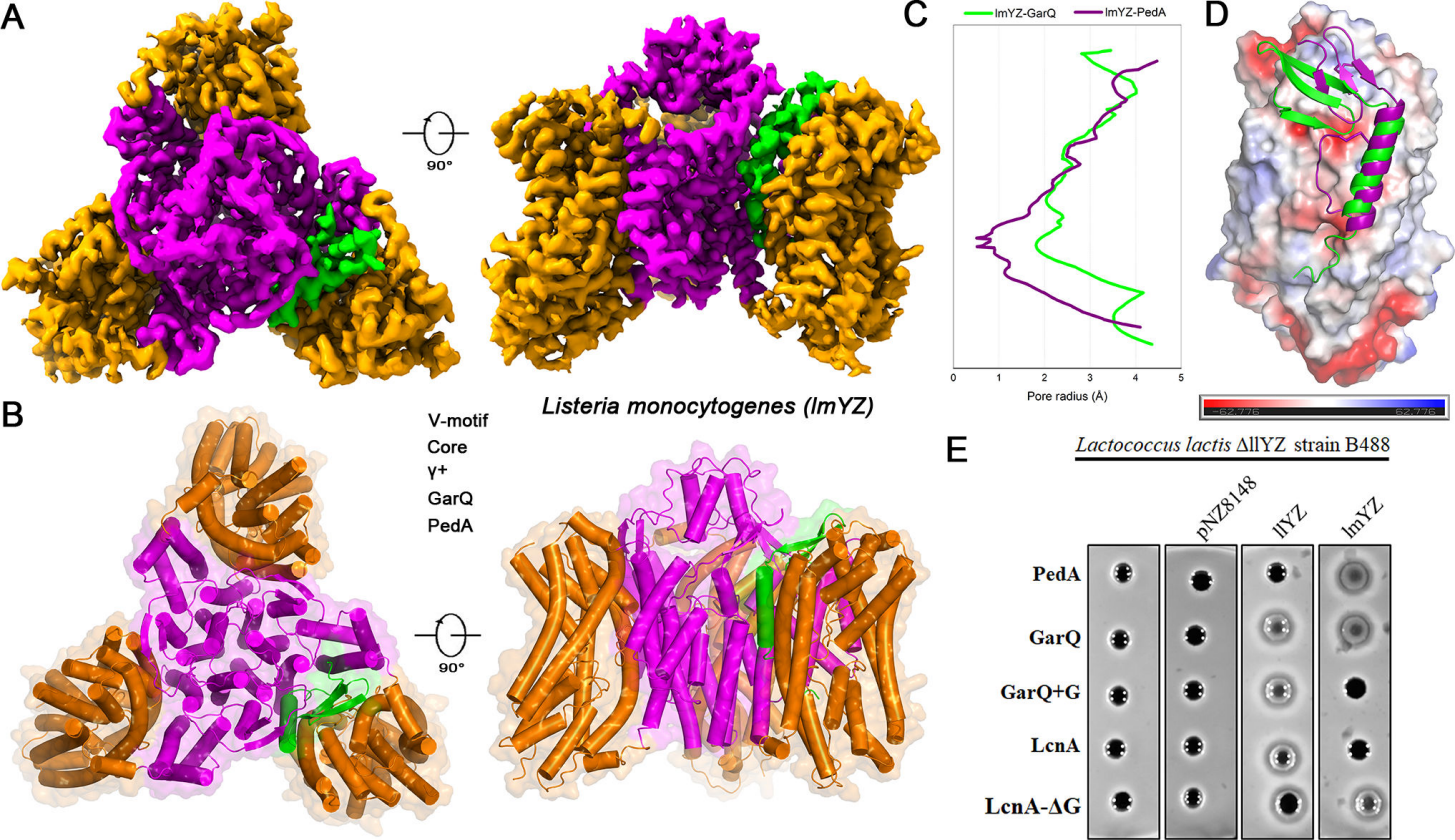
Structural basis for GarQ recognition by the Man-PTS receptor of *Listeria monocytogenes*. (**A**) Cryo-EM reconstruction of the GarQ-lmYZ complex (top and side views). (**B**) Atomic structure of the GarQ-lmYZ complex, shown in the top and side views. One GarQ monomer (green) is bound between the V-motif domain and Core domain of the lmYZ protomer. (**C**) Calculated pore radii for the channels induced by GarQ (green) and PedA (purple). The larger pore diameter for GarQ correlates with its larger molecular size. (**D**) Structural alignment of the GarQ-lmYZ (green) and PedA-lmYZ (purple; PDB ID: 7VLY) complexes, based on their Core domains. The V-motif domains were omitted for clarity. The alignment reveals that GarQ and the class IIa bacteriocin PedA bind to distinct sites on the Core domain. The molecular surface of the Core domain is shown in electrostatic potential. (**E**) Bactericidal activity assays confirming the structural model. Deletion of the C-terminal glycine from LcnA (LcnA-ΔG) enables it to target *Listeria monocytogenes*, demonstrating that C-terminal length is a key determinant of cross-species activity.

To address this, we further determined the cryo-EM structure of GarQ bound to the lmYZ (Fig. S5). Despite engaging a phylogenetically distinct receptor, GarQ retained the canonical wedge-like binding mode observed on lgYZ, inducing pore formation through an equivalent mechanism (Fig. 3A and B; Fig. S6A). The pore formed by GarQ on lmYZ was wider than that formed by PedA (PDB ID: 7VLY), reflecting GarQ’s larger size and distinct C-terminal conformation (Fig. 3C). Structural comparison revealed the basis of this promiscuity: whereas PedA binds a discrete site on the lmYZ Core domain (Fig. 3D), GarQ engages a position structurally conserved with its lactococcal binding site (Fig. S6B). Thus, rather than recognizing the species-defining α-region like native class IIa bacteriocins, GarQ exploits a conserved epitope, thereby bypassing the canonical specificity barrier.

Although GarQ and LcnA share a similar N-terminal three-stranded β-sheet, bactericidal assays demonstrated that LcnA cannot kill via the *Listeria* receptor (Fig. 3E), contradicting the expectation that absence of the γ+ domain would allow binding. This pointed to a decisive role for the C-terminus. Sequence analysis identified a single glycine insertion (G49) in LcnA’s C-terminus as the critical determinant preventing lmYZ activity (Fig. 2C). Reciprocal mutagenesis confirmed this: introducing the glycine into GarQ (GarQ+G) abolished lmYZ activity, while deleting it from LcnA (LcnA-ΔG) restored potent activity. Importantly, these manipulations did not alter bacteriocin function on the native lactococcal receptor (llYZ), highlighting the specificity of this mechanism.

Together, these results uncover a previously unrecognized strategy for extended-spectrum antimicrobial activity: preservation of a conserved binding mode across receptor orthologs. Moreover, we identify the bacteriocin C-terminus as a tunable molecular switch that defines the target spectrum, providing a rational design principle for engineering bacteriocins with tailored activity against both lactococcal and non-lactococcal pathogens.

## DISCUSSION

**Fig 4:**
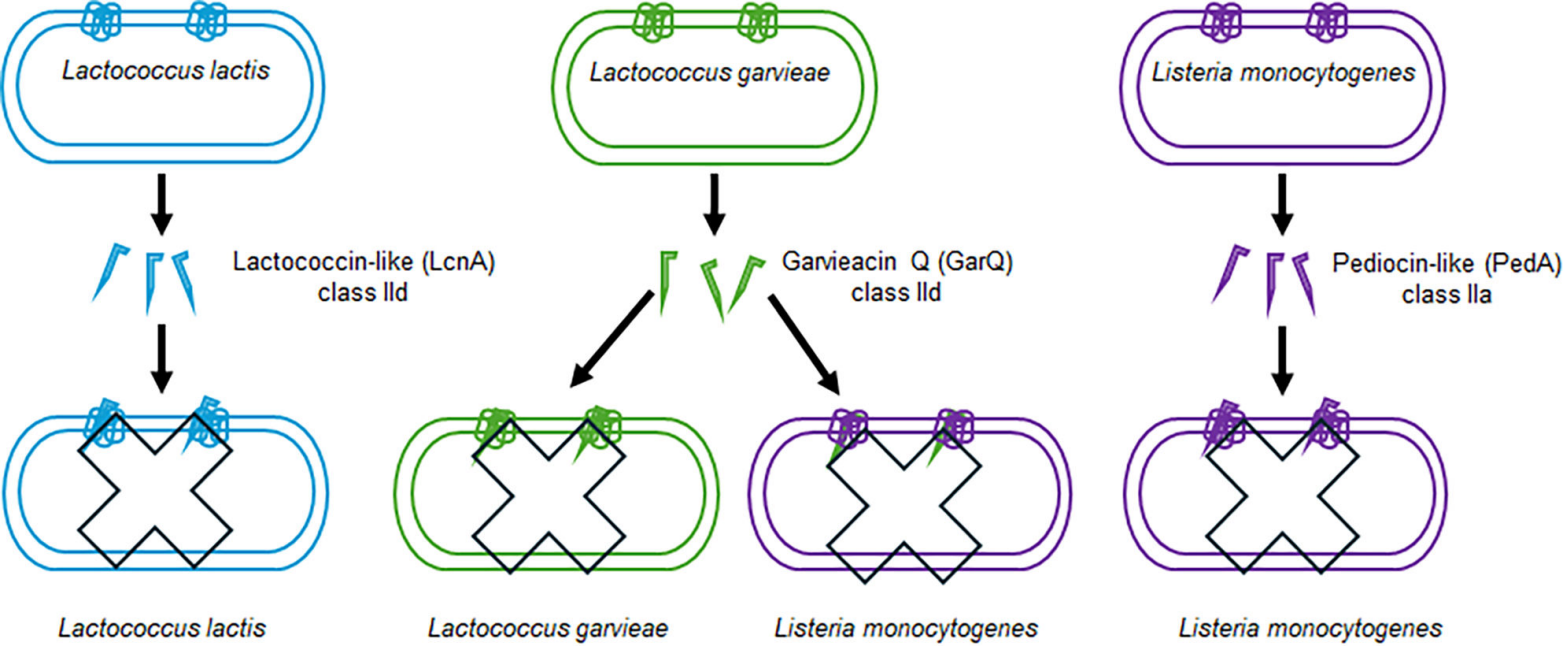
Schematic model summarizing the extended antimicrobial mechanism of GarQ. The diagram compares the target recognition and pore-forming mechanism of GarQ with those of related bacteriocins PedA and LcnA, highlighting GarQ’s extended species specificity and distinct receptor interaction.

This study defines the structural and mechanistic basis for the unusual extended-spectrum activity of GarQ, a bacteriocin that challenges the long-standing paradigm of narrow, species-specific activity within class II bacteriocins (Fig. 4). Through high-resolution cryo-EM structures of GarQ bound to its cognate Man-PTS receptors in both *Lactococcus garvieae* and *Listeria monocytogenes*, we reveal a dual mechanism: a restrictive Tudor-like domain that enforces specificity in lactococci and a conserved binding mode that enables cross-species recognition. Complementary NMR data indicate that GarQ adopts a helix-hinge-helix fold in solution (24), which was obtained in 50% TFE/water, a solvent mixture that mimics a membrane-like environment and may bias toward an all-helical conformation. This observation is not contradictory to our cryo-EM data but instead supports a model in which GarQ undergoes conformational reorganization upon receptor binding.

A defining feature of the Man-PTS receptors of *Lactococcus garvieae* is the presence of a previously uncharacterized Tudor-like γ+ domain. While Tudor domains are best known as eukaryotic modules involved in nuclear processes such as epigenetic regulation (25), their identification in a bacterial membrane transporter is unprecedented. Functionally, this accessory domain acts as a molecular gatekeeper, sterically excluding non-cognate bacteriocins such as LcnA by occluding the β2–β3 loop. This structural adaptation provides *Lactococcus garvieae* with a selective defense mechanism against closely related competitors that target the same core receptor. More broadly, our findings expand the functional repertoire of bacterial Tudor domains and illustrate how accessory modules can drastically remodel receptor specificity. Given this central role, the γ+ domain also emerges as a potential lever for modulating bacteriocin sensitivity and overcoming resistance: altering, deleting, or engineering this domain could be exploited to remodel receptor specificity and broaden the range of responsive bacteriocins.

The second major finding resolves the paradox of GarQ’s activity against *Listeria monocytogenes*. Instead of adapting to the *Listeria*-specific α-region epitope used by class IIa bacteriocins—which are broadly active not only against *Listeria* but also against closely related genera such as *Pediococcus* and *Enterococcus*—GarQ retains the canonical binding mode of its native lactococcin-A-like lineage within class IId, engaging a structurally conserved epitope on the *Listeria* receptor that is distinct from the PedA binding site. This conserved binding strategy demonstrates that, despite phylogenetic divergence, Man-PTS receptors maintain shared structural footprints that can be exploited by a promiscuous bacteriocin. Thus, GarQ represents an unusual member of the lactococcin-A-like bacteriocins (including LcnA, LcnB, and LcnZ), which are generally narrow in spectrum, but in this case exhibit extended activity across genera.

A third key insight is that bacteriocin specificity is tunable through C-terminal architecture. The single-glycine insertion in LcnA functions as an active determinant, shaping pore geometry and restricting its spectrum to lactococci. Importantly, these modifications did not compromise native activity against lactococcal receptors, indicating a modular division of labor: the N-terminus mediates conserved receptor binding, while the C-terminus functions as a spectrum-defining module. We note that while some lactococcins such as LcnG and LcnQ are classified as class IIb two-peptide bacteriocins, LcnA and its close relatives exemplify the class IId subgroup, highlighting the structural and mechanistic diversity among lactococcins.

In conclusion, our work transforms GarQ from a microbiological anomaly into a model system for both understanding and engineering antimicrobial specificity. By moving beyond the traditional one-receptor–one-bacteriocin paradigm, we reveal that specificity is multilayered: restrictive accessory domains on the receptor act as molecular gatekeepers, while modular C-terminal features of the bacteriocin serve as tunable switches that define the target spectrum. This dual-level mechanism not only reshapes our conceptual framework for bacteriocin biology but also provides a powerful foundation for applied innovation. The modularity of GarQ offers a rational strategy for synthetic biology, where accessory-domain recognition can be harnessed for strain-level precision, and C-terminal engineering can expand or narrow activity ranges to target specific pathogens. In particular, targeted manipulation of accessory domains such as γ+ provides a rational avenue to overcome emerging resistance in pathogens, while C-terminal engineering offers complementary flexibility. Together, these principles establish a blueprint for the development of “designer bacteriocins” with programmable spectra, bridging fundamental structural microbiology with translational efforts in antimicrobial therapeutics, agriculture, and food safety.

## MATERIALS AND METHODS

### Expression of lgYZ, lmYZ, and GarQ

After conducting a thorough screening for the most favorable expression conditions, it was determined that lgYZ and GarQ would be expressed using the nisin-controlled gene expression system (NICE Expression System) in *Lactococcus lactis*. On the other hand, lmYZ would be expressed utilizing the *E. coli* expression system. Genomic DNA from *Lactococcus garvieae* was extracted to obtain the original lgYZ sequence through PCR amplification. To facilitate purification, the lgYZ gene was ligated into the pNZ8148 vector via homologous recombination, with the addition of a 6× His tag at the C-terminus of lgManY. The resulting recombinant vector was then introduced into *Lactococcus cremoris* strain NZ9000 through electroporation. Cultures were incubated in a shaking incubator at 30℃ and 100 rpm. Amplification was carried out in M17 medium supplemented with 0.5% lactose and 0.05% glucose. Once the optical density at 600 nm (OD600) reached 0.5, the temperature was lowered to 18℃, and a 1 mg/mL nisin solution (200 μL per liter of culture) was added for overnight induction, approximately lasting 12 h. The GarQ gene was synthesized by a commercial company (Genewiz) and subsequently cloned into the pNZ8148 vector with an MBP tag at the N-terminus. The same expression conditions as lgYZ were employed for this process. As for lmYZ, which was also synthesized by Genewiz, codon optimization was performed for effective expression in *Escherichia coli*. It was then cloned into the pQLinkN vector, incorporating a 6× His tag at the N-terminus of lmManZ. The constructed vector was transformed into *Escherichia coli* strain C43 and cultured at 37℃ and 220 rpm until the OD600 reached 1. Subsequently, the temperature was reduced to 18℃, and 0.5 mM isopropyl β-D-thiogalactoside (IPTG) was added for overnight induction.

### Purification of lgYZ, lmYZ, and GarQ

The bacterial culture expressing the target proteins was centrifuged. Subsequently, the pellet was resuspended using a lysis buffer (50 mM MES pH 8.0, 150 mM NaCl). Cell disruption was performed using a French Press at 700–900 MPa for 4–6 cycles.

For membrane protein purification, lgYZ and lmYZ, the lysate was centrifuged at 12,000 rpm for 10 min at 4℃, and the supernatant was ultracentrifuged at 41,100 rpm for 1 h at 4℃. The membrane pellets thus obtained were collected and homogenized in lysis buffer plus 1 mM mannose and then solubilized with 2% (wt/vol) n-dodecyl-β-d-maltoside (DDM, Anatrace) at 4°C for 2 h. The insoluble fraction was precipitated by ultracentrifugation (41,100 rpm) for 30 min at 4°C. The supernatant was collected and loaded onto Ni-NTA affinity resin (Qiagen) three times and then washed with lysis buffer plus 20 mM imidazole, 0.2% DDM, and 1 mM mannose, followed by elution with lysis buffer plus 250 mM imidazole, 0.2% DDM, and 1 mM mannose.

For soluble protein GarQ’s purification, the lysate was centrifuged at 12,000 rpm for 1 h at 4℃. The supernatant was collected and loaded onto amylose resin (NEB) three times and then washed with lysis buffer, followed by elution with lysis buffer plus 1% (wt/vol) maltose.

The protein was concentrated and further applied to the Superdex 200 Increase column (GE Healthcare) re-equilibrated with buffer containing lysis buffer, 1 mM mannose, and 0.07% digitonin. The peak fractions were collected. During size-exclusion chromatography, the equilibration buffer for the soluble protein GarQ also contained a detergent (0.07% digitonin) in order to facilitate subsequent complex assembly.

### Purification of the GarQ-lgYZ and GarQ-lmYZ protein complexes

The purified ManYZ and GarQ proteins were mixed in a proportional ratio and incubated overnight at 4°C with gentle rotation. The mixture was initially subjected to Ni-NTA affinity purification, followed by purification using an amylose resin (NEB).

The complex was concentrated and further applied to the Superdex 200 Increase column (GE Healthcare) equilibrated with buffer containing lysis buffer, 1 mM mannose, and 0.07% digitonin. The peak fractions were collected.

### Cryo-EM sample preparation

The complex sample was prepared for negative staining using a 2% (wt/vol) uranyl acetate solution. Negative stain check allowed for the assessment of protein homogeneity and determination of an appropriate concentration for cryo-sample preparation. A 4 µL aliquot of the protein sample was applied onto a Cu 300 mesh with 2 nm C Quantifoil R 1.2/1.3 EM grid, which had been glow-discharged for 30 s using a Med apparatus. Subsequently, the grid was rapidly plunge-frozen in liquid ethane, pre-cooled with liquid nitrogen, using the Thermo Fisher Scientific Mark IV Vitrobot device. The grid was blot-dried for 3.5 s after a 20 s waiting period and then flash-frozen in liquid ethane cooled by liquid nitrogen with a Vitrobot Mark IV (FEI) at 100% humidity and 8°C.

### EM data acquisition and image processing for lgYZ, lgYZ-GarQ, and lmYZ-GarQ

Data collection included 3,325 movies for the lgYZ, 6,048 movies for the lgYZ-GarQ complex, and 3,384 movies for the lmYZ-GarQ complex. All of the movies were performed using a 300 kV Titan Krios electron microscope (Thermo Fisher) fitted with a K3 Summit counting camera (Gatan), with a pixel size of 1.0742. Each movie contained 32 frames and received an approximate total dose of 50 e^-^/Å^2^, with defocus values ranging between −1.5 and −1.8 µm. Data collection was fully automated, facilitated by AutoEMation software (26). Motion correction was performed using MotionCor2 v1.2.6 (27), while GCTF v.1.18 (28) was used for contrast transfer function (CTF) estimation. Micrographs with a CTF fitting resolution worse than 6 Å were discarded. All subsequent processing steps were conducted using cryoSPARC (29).

### Protein model building and structure refinement

Protein models were built *de novo* in EMBuilder (30) and subsequently manually adjusted using UCSF Chimera (31) or COOT (32). Structural refinement was performed using PHENIX (33). Table S1 provides statistical details regarding the 3D reconstruction and model refinement processes. Visual representations of the structures were generated using PyMol (34).

### Expression and purification of PedA, LcnA, GarQ, and their variants

Recombinant PedA, LcnA, GarQ, and their variants were expressed in *E. coli* C43 (DE3). Bacterial cultures were grown at 37°C to an OD600 of 0.8, at which point, the expression was induced with IPTG and continued overnight at 18°C. Cells were harvested by centrifugation, resuspended in lysis buffer (50 mM MES, pH 6.5, 150 mM NaCl), and lysed via sonication. Following centrifugation at 12,000 rpm for 1 h at 4°C, the supernatant was incubated with amylose affinity resin. The resin was washed extensively, and the bound bacteriocins were eluted with the appropriate buffer.

### Bacteriocin activity assay

Bacteriocin activity was assessed using a plate diffusion assay. Various ManYZ constructs were cloned into the pNZ8148 vector and electroporated into competent *Lactococcus lactis* B488 cells (ΔllYZ). The resulting recombinant strains were cultured at 30°C for 4 h, after which protein expression was induced with 200 ng/mL nisin at 18°C for 16 h. Cells were normalized to an OD600 of 1.0, mixed with M17 medium containing 0.7% agar, and then overlaid onto 10 cm dish. Wells were created in the solid bacteria-containing medium and filled with 80 μL of each bacteriocin solution. The plates were incubated at 30°C for 12 h, and the resulting zones of inhibition were observed.

## Data Availability

Cryo-EM maps and the associated structural coordinates, respectively, have been deposited into the Electron Microscopy Data Bank (EMDB) and the Protein Data Bank (PDB) under the following accession codes: EMD-66027/9WJR (lgYZ), EMD-66030/9WJU (GarQ-lgYZ), and EMD-66032/9WJW (GarQ-lmYZ).

## SUPPLEMENTAL MATERIAL

Supplemental material aem.01773-25-s0001.docx Figures S1 to S6; Table S1.

ASM does not own the copyrights to Supplemental Material that may be linked to, or accessed through, an article. The authors have granted ASM a non-exclusive, world-wide license to publish the Supplemental Material files. Please contact the corresponding author directly for reuse.
