# Supplementary material for: Structural basis for the extended-spectrum antimicrobial activity of Garvieacin Q
Source: Appl Environ Microbiol. 2026 Jan 21;92(2):e01773-25. doi: 10.1128/aem.01773-25 (PMC12915331; doi:10.1128/aem.01773-25)
Supplement: Supplemental material — Figures S1 to S6; Table S1. [file aem.01773-25-s0001.docx]

Supplementary information for

Structural basis for the extended-spectrum antimicrobial activity of Garvieacin Q

This PDF file includes:

Supplementary Figures S1 to S6

Supplementary Table S1

# Supplementary Figures


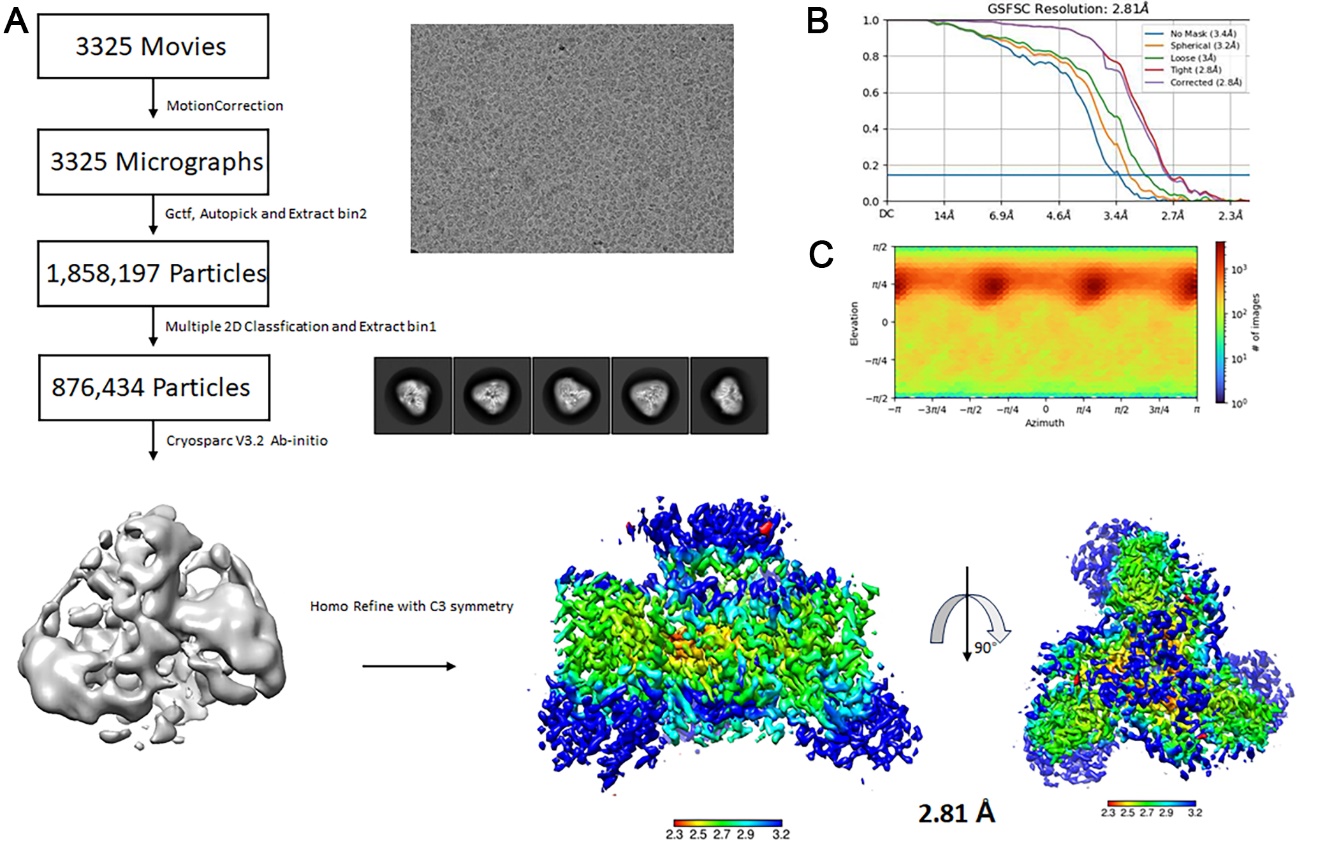


1. **Cryo-EM data processing and reconstruction for the *Lactococcus garvieae* Man-PTS (lgYZ).**
2. Workflow diagram of the cryo-EM processing pipeline for lgYZ. Local resolution map of the final 3D reconstruction is shown, with resolution values indicated by the color scale (Å) (1).
3. Gold-standard Fourier shell correlation (FSC) curve showing the overall resolution of the final map (FSC = 0.143 indicated by the horizontal line) (2).
4. Angular distribution plot of particle orientations used in the final 3D reconstruction.


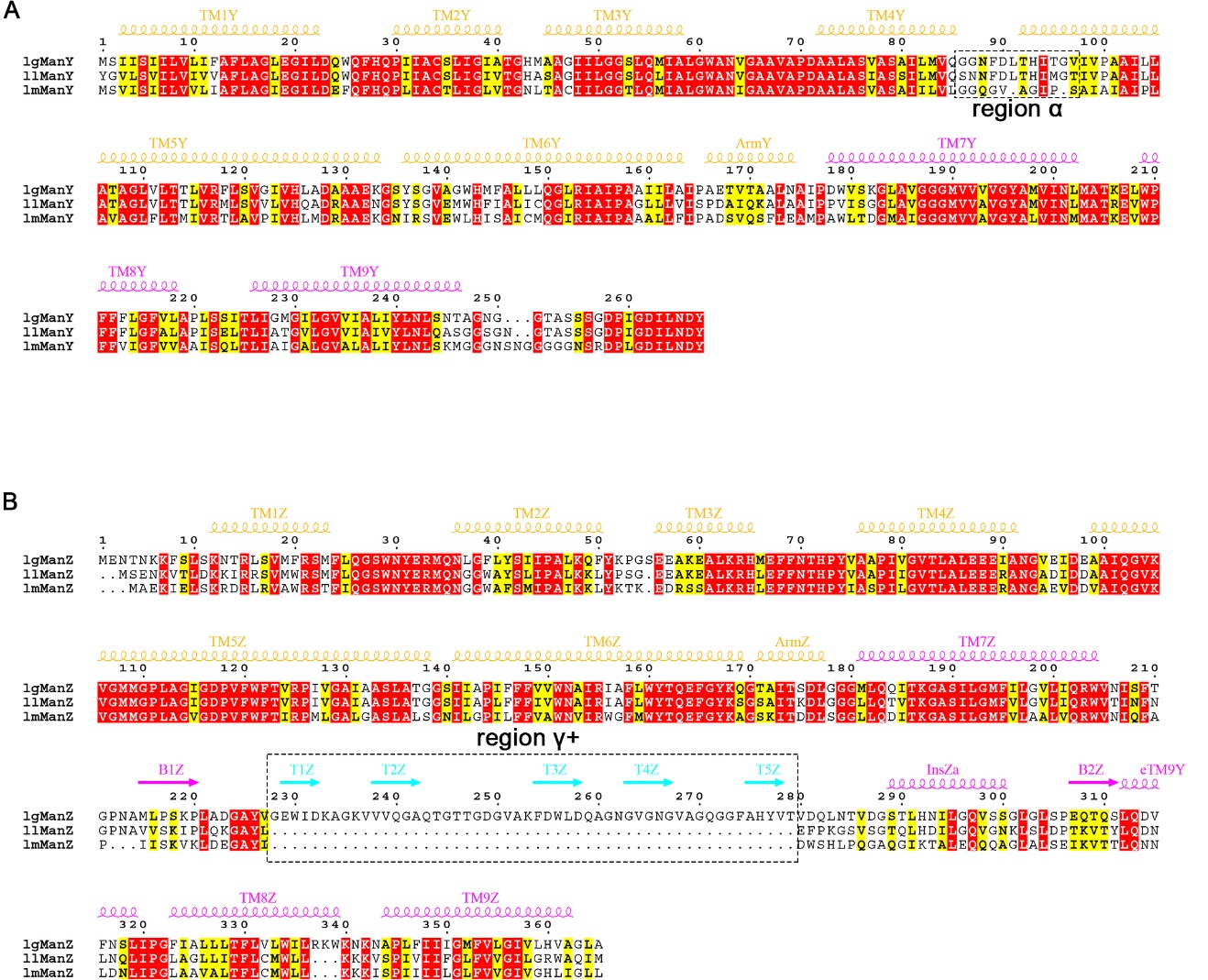


1. **Sequence alignment of ManY and ManZ subunits.**
2. Alignment of ManY from *Lactococcus garvieae* (lgManY), *Lactococcus lactis* (llManY), and *Listeria monocytogenes* (lmManY), with secondary structure elements of lgManY indicated above.
3. Alignment of ManZ from the same species. Secondary structure of lgManZ shown above, and the unique γ+ insertion is highlighted.


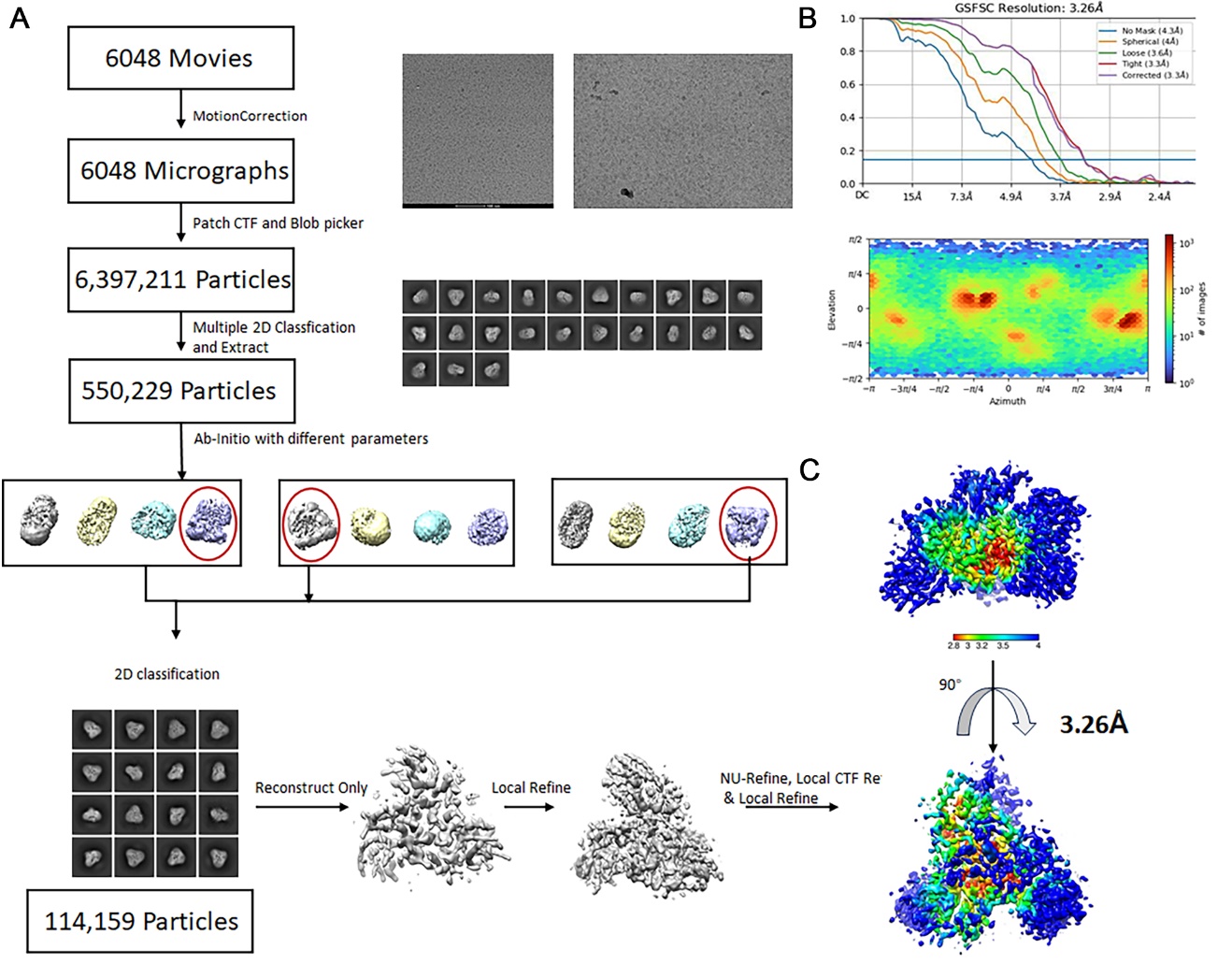


1. **Cryo-EM data processing and reconstruction of lgYZ-GarQ complex.**
2. Workflow diagram of the cryo-EM processing pipeline for lgYZ-GarQ. Local resolution map of the final 3D reconstruction is shown with resolution values (Å).
3. Gold-standard FSC curve indicating the overall resolution (FSC = 0.143 shown).
4. Angular distribution of particle orientations used for 3D reconstruction.


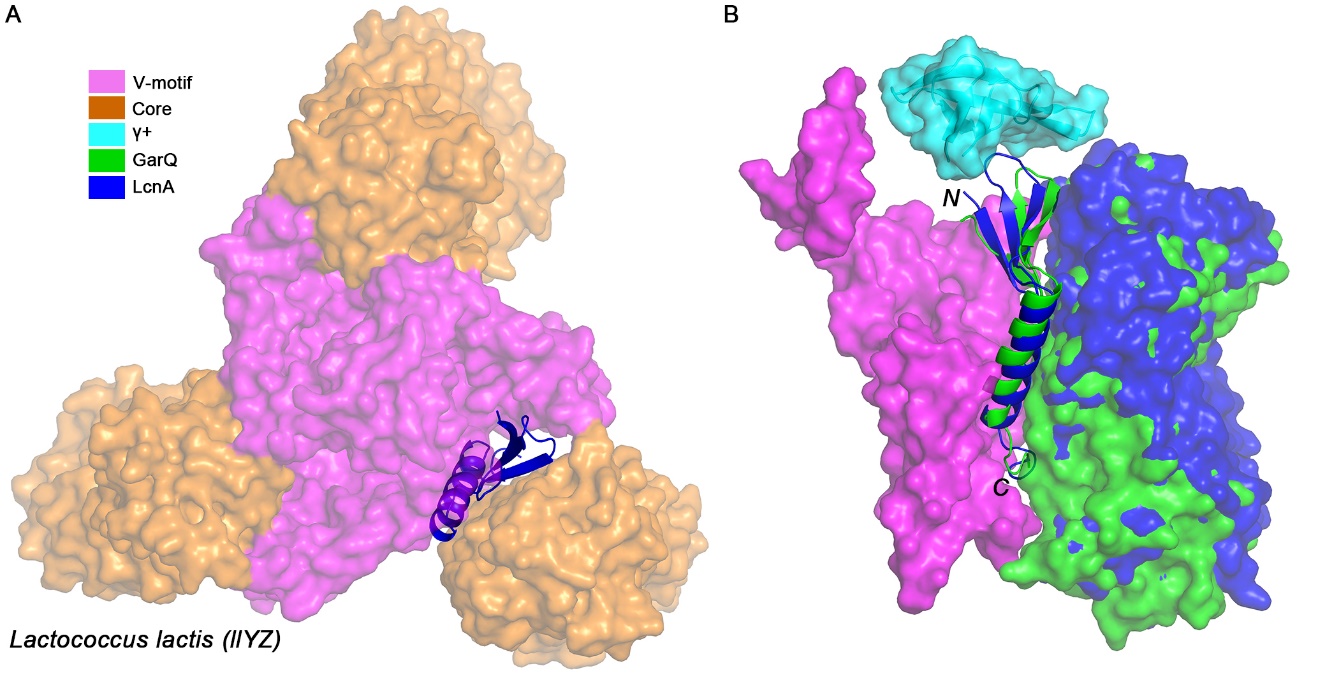


1. **Structural comparison of class IId bacteriocin-receptor complexes.**
2. Top view of the cryo-EM structure of the llYZ Man-PTS bound to LcnA (blue).
3. Structural alignment of GarQ-lgYZ and LcnA-llYZ complexes based on V-motif domains. Core domains and bound bacteriocins retain their respective colors. Divergence in Core domain orientation, largely influenced by bacteriocin C-termini, is highlighted.


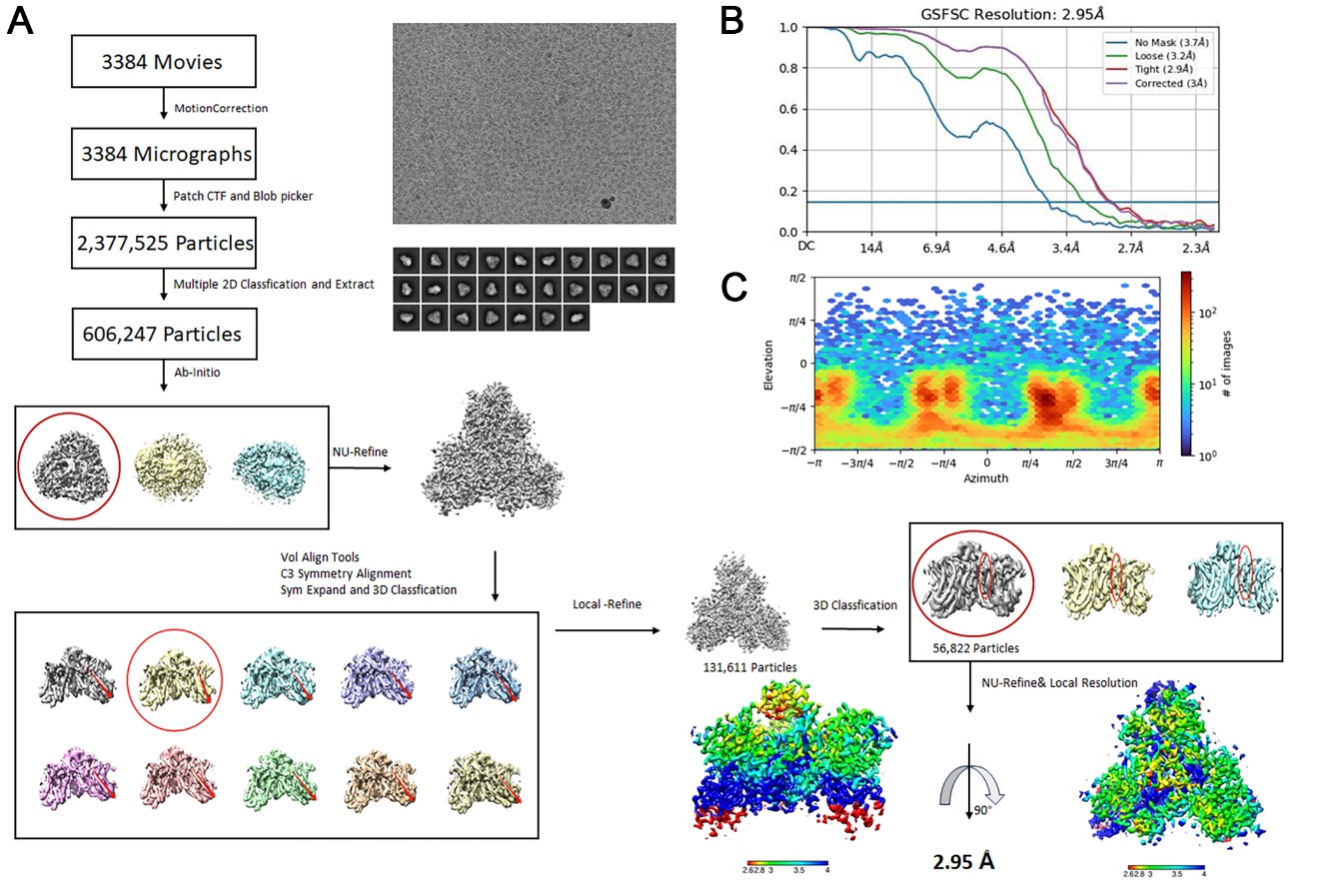


1. **Cryo-EM data processing and reconstruction of lmYZ-GarQ.**
2. Workflow diagram of the cryo-EM processing pipeline for lmYZ-GarQ. Local resolution map of the final 3D reconstruction is shown (Å).
3. Gold-standard FSC curve showing overall resolution (FSC = 0.143 indicated).
4. Angular distribution of particle orientations used for 3D reconstruction.


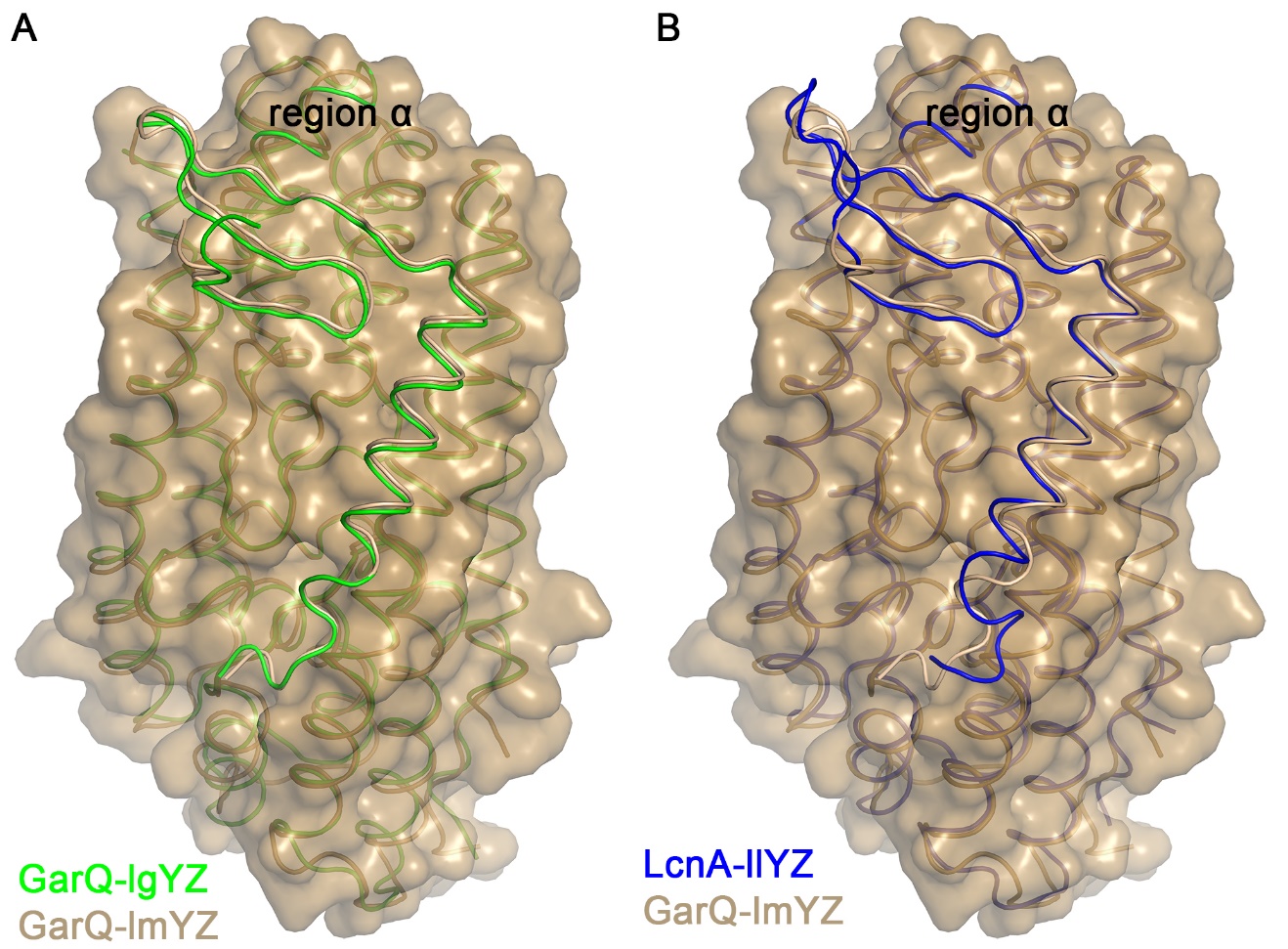


1. **Structural alignment of bacteriocin-receptor complexes based on Core domain.**
2. Superposition of GarQ-lgYZ (green) and GarQ-lmYZ (wheat) complexes. High structural conservation of GarQ’s binding mode across receptor subtypes demonstrates its versatile, conserved mechanism.
3. Superposition of LcnA-llYZ (blue) and GarQ-lmYZ (wheat) complexes. Alignment reveals that GarQ engages the non-cognate *Listeria* receptor using a binding pose closely resembling the canonical LcnA-lactococcal interaction, explaining its cross-species activity.

# Supplementary Tables

1. **Cryo-EM data collection, refinement and validation statistics**

|  | lgYZ | GarQ-lgYZ | GarQ-lmYZ |
| --- | --- | --- | --- |
| Voltage (kV) | 300 | 300 | 300 |
| Electron exposure (e^-^/Å^2^) | 50 | 50 | 50 |
| Defocus range (μm) | -1.5 ~ -1.8 | -1.5 ~ -1.8 | -1.5 ~ -1.8 |
| Pixel size (Å) | 1.0742 | 1.0742 | 1.0742 |
| Micrographs (no.) | 3,325 | 6,048 | 3,384 |
| Symmetry imposed | C3 | C1 | C1 |
| Final particle images (no.) | 876,434 | 114,159 | 56,822 |
| Map resolution (Å) 0.143 FSC threshold | 2.81 | 3.26 | 2.95 |
| Map sharpening B factor (Å^2^) | -124.1 | -86.2 | -88.6 |
| CC (model vs. data) | 0.8050 | 0.7758 | 0.8419 |
| Model composition |  |  |  |
| Chain count | 6 | 7 | 7 |
| Non-hydrogen atoms | 13,605 | 14,017 | 12,736 |
| Protein residues | 1,821 | 1,826 | 1,639 |
| Bacteriocin residues | - | 51 | 50 |
| B factors (Å^2^) |  |  |  |
| Proteins | 65.99 | 147.71 | 86.47 |
| Mannose | 56.08 | 144.54 | 73.99 |
| Bacteriocins | - | 123.68 | 91.47 |
| R.m.s. deviations |  |  |  |
| Bond lengths (Å) | 0.004 | 0.003 | 0.003 |
| Bond angles (°) | 0.622 | 0.677 | 0.638 |
| Validation |  |  |  |
| MolProbity score | 1.83 | 2.07 | 2.53 |
| Clashscore | 7.7 | 13.04 | 11.53 |
| Poor rotamers (%) | 0.72 | 0.28 | 4.66 |
| Ramachandran plot |  |  |  |
| Favoured (%) | 93.92 | 93.08 | 93.13 |
| Allowed (%) | 5.75 | 6.71 | 6.69 |
| Disallowed (%) | 0.33 | 0.21 | 0.18 |
| PDB code | 9WJR | 9WJU | 9WJW |
| EMDB code | EMD-66027 | EMD-66030 | EMD-66032 |

# References

1. Kucukelbir A, Sigworth FJ, Tagare HD. 2014. Quantifying the local resolution of cryo-EM density maps. Nat Methods 11:63-5.

2. Rosenthal PB, Henderson R. 2003. Optimal determination of particle orientation, absolute hand, and contrast loss in single-particle electron cryomicroscopy. J Mol Biol 333:721-45.
